# Transcriptome analysis of highly purified mouse spermatogenic cell populations: gene expression signatures switch from meiotic-to postmeiotic-related processes at pachytene stage

**DOI:** 10.1186/s12864-016-2618-1

**Published:** 2016-04-19

**Authors:** Irene da Cruz, Rosana Rodríguez-Casuriaga, Federico F. Santiñaque, Joaquina Farías, Gianni Curti, Carlos A. Capoano, Gustavo A. Folle, Ricardo Benavente, José Roberto Sotelo-Silveira, Adriana Geisinger

**Affiliations:** Department of Genomics, Instituto de Investigaciones Biológicas Clemente Estable (IIBCE), Av. Italia 3318, 11,600 Montevideo, Uruguay; Department of Molecular Biology, Instituto de Investigaciones Biológicas Clemente Estable (IIBCE), Av. Italia 3318, 11,600 Montevideo, Uruguay; Flow Cytometry and Cell Sorting Core, IIBCE, Montevideo, Uruguay; Department of Proteins and Nucleic Acids, IIBCE, Montevideo, Uruguay; Department of Genetics, IIBCE, Montevideo, Uruguay; Department of Cell and Developmental Biology, Biocenter, University of Würzburg, D-97074 Würzburg, Germany; Department of Cell and Molecular Biology, Facultad de Ciencias, Universidad de la República (UDELAR), 11,400 Montevideo, Uruguay; Biochemistry-Molecular Biology, Facultad de Ciencias, UDELAR, Montevideo, Uruguay

**Keywords:** Spermatogenesis, Transcriptome, RNAseq, Flow cytometry

## Abstract

**Background:**

Spermatogenesis is a complex differentiation process that involves the successive and simultaneous execution of three different gene expression programs: mitotic proliferation of spermatogonia, meiosis, and spermiogenesis. Testicular cell heterogeneity has hindered its molecular analyses. Moreover, the characterization of short, poorly represented cell stages such as initial meiotic prophase ones (leptotene and zygotene) has remained elusive, despite their crucial importance for understanding the fundamentals of meiosis.

**Results:**

We have developed a flow cytometry-based approach for obtaining highly pure stage-specific spermatogenic cell populations, including early meiotic prophase. Here we combined this methodology with next generation sequencing, which enabled the analysis of meiotic and postmeiotic gene expression signatures in mouse with unprecedented reliability. Interestingly, we found that a considerable number of genes involved in early as well as late meiotic processes are already on at early meiotic prophase, with a high proportion of them being expressed only for the short time lapse of lepto-zygotene stages. Besides, we observed a massive change in gene expression patterns during medium meiotic prophase (pachytene) when mostly genes related to spermiogenesis and sperm function are already turned on. This indicates that the transcriptional switch from meiosis to post-meiosis takes place very early, during meiotic prophase, thus disclosing a higher incidence of post-transcriptional regulation in spermatogenesis than previously reported. Moreover, we found that a good proportion of the differential gene expression in spermiogenesis corresponds to up-regulation of genes whose expression starts earlier, at pachytene stage; this includes transition protein-and protamine-coding genes, which have long been claimed to switch on during spermiogenesis. In addition, our results afford new insights concerning X chromosome meiotic inactivation and reactivation.

**Conclusions:**

This work provides for the first time an overview of the time course for the massive onset and turning off of the meiotic and spermiogenic genetic programs. Importantly, our data represent a highly reliable information set about gene expression in pure testicular cell populations including early meiotic prophase, for further data mining towards the elucidation of the molecular bases of male reproduction in mammals.

**Electronic supplementary material:**

The online version of this article (doi:10.1186/s12864-016-2618-1) contains supplementary material, which is available to authorized users.

## Background

Spermatogenesis is a very complex terminal cell differentiation process that yields mature sperm, and results from the expression of a specific genetic program [1, 2]. It has been divided into three succesive phases: somatic proliferation of spermatogonia, meiosis, and spermiogenesis. Each of these phases is characterized by its own gene expression program, and all the phase-specific programs are executed in the testis in a simultaneous and coordinated way.

In the seminiferous tubules of adult mammals, cell types with different DNA content coexist: several types of G1 phase spermatogonia and secondary spermatocytes (2C), various stages of primary spermatocytes and G2 phase spermatogonia (4C), different steps of round and elongating spermatids (C), and spermatozoa (C). Besides, somatic Sertoli cells (2C) also reside inside the tubules, totaling over 30 diverse coexistent cell types. Moreover, seminiferous tubules are surrounded by peritubular myoid cells and immersed in a stroma containing fibroblasts, lymphocytes, mastocytes, macrophages, and Leydig cells, all of them 2C.

The heterogeneity of testicular cell composition together with the lack of *in vitro* systems for spermatogenic cell culture [3] have been important drawbacks for gene expression studies along the different spermatogenic stages. Basically two approaches have been used in order to overcome these limitations. The first approach has been the analysis of RNA from whole testes of prepubertal animals at different ages representative of the first spermatogenic wave progression (*e.g*. [4–11]), where the transcripts that are present at one age but not at younger ages are attributed to the newly appeared cell types. However, this strategy does not allow undoubtedly assigning specific RNAs to a certain cell type within the heterogeneous population. Moreover, specific transcripts from very poorly represented cell types will probably escape detection, as they appear diluted in comparison to highly represented ones; even abundant transcripts from scanty cell types are usually hidden. Other than that, although it has been asserted that in rodents the first spermatogenic wave is synchronous [6, 7, 11], we and others [12] have observed that this is not entirely true and therefore partial desynchronization will also contribute to wrong conclusions.

A second strategy has been the enrichment of stage-specific cell populations. Among separation techniques, the most widely used have been gravimetric decantation (Staput) [13–15] and centrifugal elutriation [16]. Due to the relative abundance and cell size difference, these methodologies only allow the successful separation of highly enriched fractions containing pachytene spermatocytes (PS; *i.e*., medium meiotic prophase), or round spermatids (RS; *i.e*., spermiogenesis), while other cell types are obtained at very low purity levels [16]. Even for PS and RS-enriched cell populations, purity levels are at best around 80 % ([16], and our own experience). As a consequence, highly sensitive modern analysis techniques (mostly PCR-based) can generate misleading results caused by the amplification of transcripts from contaminating cell types. A different methodological approach, flow cytometry (FCM), has been employed to analyze and sort different testicular cell populations with unprecedented purity levels based on DNA content together with differences in nuclear size, cellular size, complexity, and chromatin compaction [12, 17–20]. Some other strategies such as laser-capture microdissection of specific spermatogenic stages [21] are very complex and require a highly experienced operator [22], and therefore have been only adopted in a limited number of laboratories (*e.g*. [23]).

The molecular characterization of meiotic prophase I early stages (leptotene and zygotene; LZ) is essential towards understanding the fundamentals of meiosis: mutations of meiotic genes that disrupt bouquet formation, chromosome alignment, meiotic synapsis, and recombination (crossing-over) are not able to reach the pachytene stage (P) often resulting in sterility [24–26]. However, the relatively short duration of these stages and consequently their low frequency in most rodent models has hampered their efficient purification [19, 20].

We have reported the sorting of LZ stages by FCM at very high purity levels (98 %), first in guinea pig (*Cavia porcellus*), a rodent that exhibits an unusually high representation of early meiotic cell populations [19], and more recently in mouse [27]. In the latter we used a DNA-specific, non-cytotoxic, vital dye (Vybrant DyeCycle Green [VDG]) that allowed an efficient discrimination of different meiotic stages (LZ from PS) as discrete populations in the dot plots. This dye is excited at 488 nm, unlike bisbenzimidazole stains such as Hoechst that require a UV laser (reviewed in [28]). We have employed flow sorting using VDG in combination with an ultra-fast method for the preparation of testicular cell suspensions developed by our group, which renders excellent preparations while avoiding the use of enzymatic treatments [29, 30]. The speed of the cell suspension preparation together with the lack of enzyme treatments helps preserve delicate macromolecules such as mRNAs, while the avoidance of UV light exposure minimizes potential damage to nucleic acids.

During the past decade several large scale differential gene expression studies along spermatogenesis have been performed in rodents, most of them based on microarray technology and starting either from whole testes of prepubertal animals [4–10] or elutriation/Staput-enriched cell populations (mainly PS and RS) [23, 31–34]. Besides, a report by Fallahi et al. [35] has employed fluorescence-activated cell sorting (FACS) to isolate meiotic cell stages for microarray expression analysis. Microarray studies have identified an important number of candidate genes for roles in the regulation of fertility and possible contraceptive targets (*e.g*. [4, 33]). Nonetheless, it has become evident that this technology is not the most suitable for testis-addressed gene expression studies as most microarrays are limited to known transcripts, and the sequences used for their construction do not contemplate testicular peculiarities such as the remarkable number of splice variants that are differentially expressed in spermatogenic cells (*e.g*. [36, 37]) .

Recently, next generation sequencing (NGS) has been applied to gene expression analysis along spermatogenesis. Besides its better sensitivity, NGS has the capability to identify and quantify novel transcribed regions and splice variants [11, 38, 39]. Two reports published hitherto have analyzed the fluctuations of gene expression patterns along spermatogenesis using whole testes at different time points during the first wave of murine spermatogenesis [11, 39]. In one of these reports, a computational deconvolution approach has been coupled as an attempt to estimate cell type-specific gene expression [39]. A handful of studies have employed elutriation-enriched testicular cell populations from mouse [38, 40] and rat [41], although they mainly focused on a global analysis of testis transcriptome complexity and chromatin changes [38, 40], or dealt with non-coding RNAs [41]. In these studies PS were used as the representative meiotic stage and no information regarding LZ was provided (as stated above, elutriation does not allow to purify LZ). On the other hand, an RNAseq study aimed at the analysis of gene expression differences between the wild type and a knockout mouse included a cell fraction modestly enriched in LZ by Staput [42]. So far, no transcriptomic studies using FCM for high purity sorting of testicular cell populations together with RNAseq have been published.

Here, we report on a detailed analysis of gene expression patterns along mouse spermatogenesis by NGS. For the present study, we have taken advantage of the above-mentioned protocols for ultra-fast preparation of testicular cell suspensions and high purity sorting with VDG, which rendered high quality, cell-type specific RNA for transcriptomic studies. The combined use of flow sorting and NGS, both extremely robust methodologies, allowed to obtain results concerning differential gene expression in the different spermatogenic stages with unprecedented reliability. Moreover, this is the first time that a highly purified LZ cell population is included in NGS studies. Remarkably, with this experimental approach we have been able to disclose gene core signatures regarding the meiotic and postmeiotic expression patterns, thus providing new insights into spermatogenesis gene regulation.

## Results

### RNAseq of highly purified testicular cell populations

In order to reliably assess gene expression changes between the different spermatogenic stages, we performed transcriptome analysis of FCM highly purified cell populations representative of landmark points along mouse spermatogenesis: 2C, LZ, PS, and RS.

**Fig. 1:**
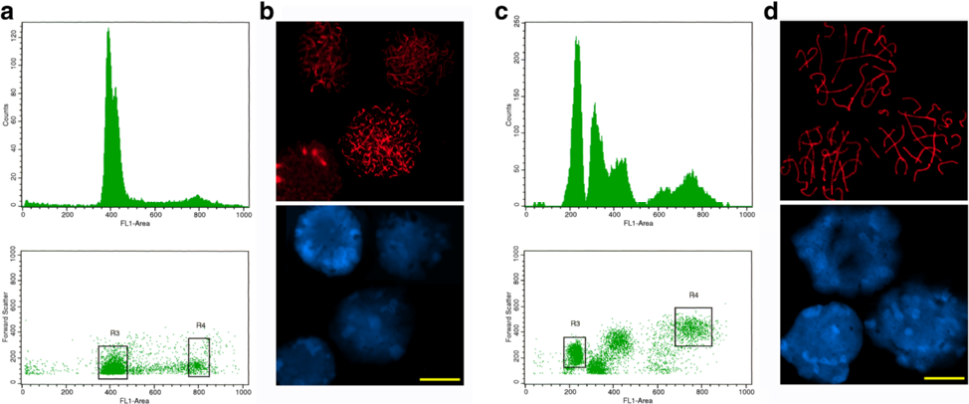
Flow cytometric purification and immunocytochemical analysis of sorted testicular cell populations with VDG. Mice aging 10–11 dpp (**a**, **b**) and 24–25 dpp (**c**, **d**) were used. **a**, **c**. Dot plots depicting forward scatter (FSC-H) *vs* VDG fluorescence intensity and their corresponding histograms showing the gated cell populations. **b**, **d**. Confocal immunocytochemical analysis with anti-SYCP3 antibody (red) as a marker of the LZ (**b**) and PS (**d**) sorted fractions. Nuclei were counterstained with DAPI. Bars correspond to 10 μm

The optimal ages for obtaining the different cell fractions (maximum representation of each specific cell type, lowest proportion of possible contaminant cell types) were determined by monitoring the progress of the first spermatogenic wave by FCM analysis of the cell suspensions, and by microscopic observation of Epon-embedded cross sections of seminiferous tubules (Additional file 1: Figure S1A). Besides, meiotic prophase stages were scrutinized by confocal immunocytochemistry using an antibody against the main component of the lateral element of synaptonemal complexes, SYCP3 (Additional file 1: Figure S1B). Accordingly, we used 10–11 days postpartum (dpp) mice for purifying the highest amount of LZ spermatocytes without any PS contamination (Fig. 1a and b). Testes from different individuals of the same age were employed for the purification of the 2C fraction, which consists of a heterogeneous cell population containing spermatogonia and somatic cells (mostly Sertoli cells), and was used as a reference for the transcripts present in pre-meiotic and somatic testicular cell types. Besides, the choice of early prepubertal mice for the purification of the 2C fraction avoids the presence of spermatocytes II, which otherwise would co-purify with this fraction due to their DNA content.

PS were obtained from the testes of 24–25 dpp pups, which showed a relatively high representation of this cell type in the seminiferous tubules. Although the 4C fraction at that age also contains L and Z spermatocytes, the use of VDG stain allowed to clearly discriminate two sub-peaks within this fraction, as follows (Fig. 1c): the leftmost 4C peak corresponded to spermatocytes in LZ stages and the rightmost one only contained PS, as shown by SYCP3 staining pattern (Fig. 1d; see also [27]). The visualization of PS as a separate, discrete population in the dot plots (see Fig. 1c) enabled its purification. Testes from individuals of the same age were employed for the purification of the C cell population. Despite the fact that a few elongating spermatids are also present at that age [17], the RS cell population was sorted without any detectable contamination from elongating spermatids. All four cell populations were obtained with 98 % purity, as assessed by FCM re-analysis and immunocytochemical studies of the sorted fractions.

RNAs from the four purified cell populations were linearly amplified with the Ovation RNA-Seq System v2 in order to increase the yield without losing RNAs complexity [43], and subjected to Illumina sequencing. Total number of reads for each sample varied from 48 to 65 million, and the mapping rate of the reads was 56-80 % (Additional file 1: Table S1).

**Fig. 2:**
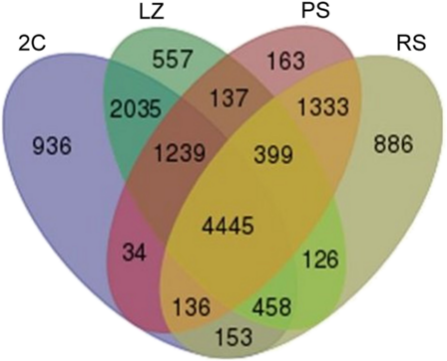
Venn diagram showing all the expressed genes in 2C, LZ, PS, and RS cell populations. Separate and overlapping expression between samples are shown. Only transcripts with a level of expression of RPKM ≥ 2 were considered

Using a high stringency (minimum read count of ≥10), a total of 13,037 expressed protein-coding genes were identified only considering genes with ≥2 reads per kilobase per million mapped reads (RPKM) in at least one of the four populations. We identified 9,436 expressed genes in the 2C population, 9,396 in LZ, 7,886 in PS, and 7,936 in RS. Among all the detected genes, 4,445 were shared by the four samples (Fig. 2).

### Major differences in gene expression patterns between LZ and PS cell populations

We performed pairwise differential gene expression comparisons between cell populations in chronological order (2C *vs* LZ, LZ *vs* PS, PS *vs* RS) to detect the genes that changed their expression by at least 2-fold (Kal’s test *p* ≤ 0.01).

**Fig. 3:**
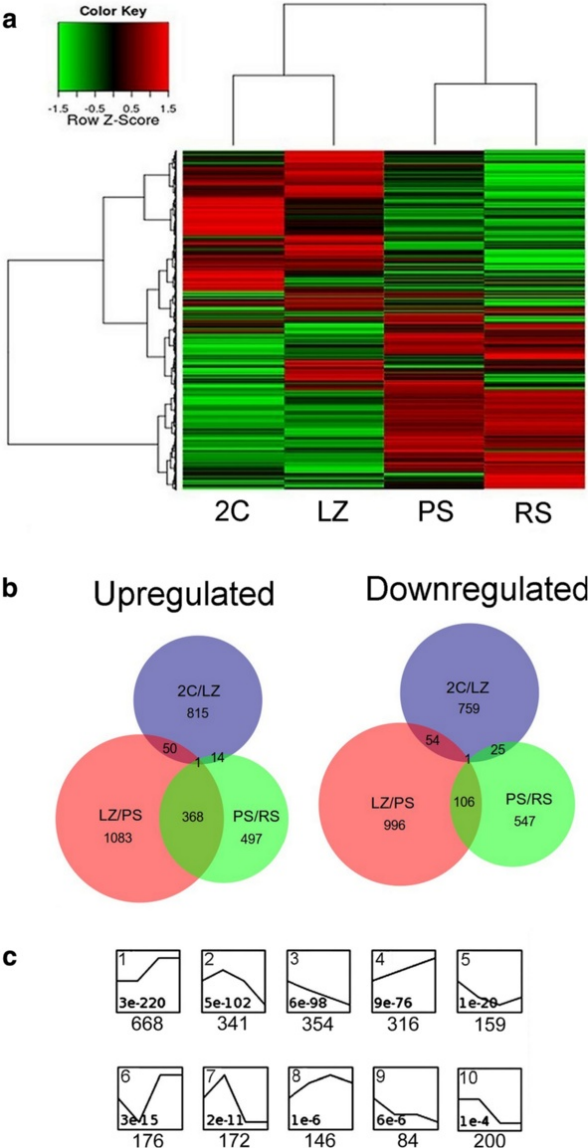
Representation of DEG between pairwise sample comparisons of the four populations in chronological order. The following comparisons were performed: 2C/LZ; LZ/PS; PS/RS (|FC| ≥ 2; Kal’s test *p* ≤ 0.01). **a**. Heat map of expression levels and hierarchical clustering for the global gene expression in the four samples. All genes detected as differential in at least one sample were included. Z-score values are coded on the green-to-red scale (high expression: red; low expression: green). **b**. Venn diagram of up-regulated and down-regulated genes. Separate and overlapping expression between samples is shown. **c**. Temporal expression profiles of DEG, ordered based on the *p*-value significance of the number of assigned *vs* expected genes. Only the 10 most significant profiles are shown. The *p*-value (bottom of each panel) and number of genes (below) for each profile are shown

Hierarchical clustering of differentially expressed genes (DEG) showed relatively similar patterns between the 2C and LZ populations on one side, and between the PS and RS populations on the other. Moreover, the global pattern of turned on/turned off genes appeared practically reversed between the LZ and PS cell populations (Fig. 3a). Accordingly, the highest number of DEG was found when comparing LZ and PS cell populations, both for up-regulated and for down-regulated genes (Table 1 and Fig. 3b). In addition, as illustrated in Fig. 3c, temporal expression profile analysis of the DEG showed that the highest number of genes under a single profile (*n* = 668) pertains to genes that are up-regulated between LZ and PS (profile 1).

**Table 1:** Total numbers of up-regulated and down-regulated genes in pairwise comparisons

Samples (comparison)	Total	UP	DOWN
2C/LZ^*a*^	1,719	880	839
LZ/PS^*a*^	2,659	1,502	1,157
PS/RS^*a*^	1,559	880	679

^*a*^Values correspond to the total, up, and down-regulated genes in group 2 relative to group 1 for each comparison (LZ relative to 2C; PS relative to LZ; RS relative to PS)

The different testicular cell populations were compared according to their temporal appearance along the spermatogenic wave (|FC| ≥ 2; *p* ≤ 0.01)

Next, we evaluated the number of up-regulated genes that were *de novo* turned on between comparisons. While 1451 of the 1502 up-regulated genes in the LZ/PS comparison were *de novo* turned on in PS, only 497 of the 880 up-regulated genes in RS were newly expressed, thus indicating that almost half of the up-regulated genes in RS correspond to increased expression (fold change [FC] ≥2) of genes already up-regulated in PS (see Fig. 3b). This shows that the P stage not only presents the highest number of up-regulated genes, but also the largest amount of *de novo* expressed genes of all the analyzed cell populations.

### A high number of meiosis-related genes are differentially expressed during early prophase

The experimental protocol employed here rendered highly pure LZ cell populations. Thus, we were able to perform a detailed transcriptome analysis of the LZ stage, and compare it with previous and following stages. In mouse, L and Z together last in total 2–3 days. For this cell population, we found 880 up-regulated and 839 down-regulated genes (see Table 1). As expected, all seven genes coding for synaptonemal complex proteins (*i.e. Sycp1*, *Sycp2*, *Sycp3*, *Syce1*, *Syce2*, *Syce3*, and *Tex12*) and key genes involved in initial recombination events (*e.g. Spo11*, *Dmc1*, *Rad51*) [44] up-regulate in LZ (see Additional file 2: Dataset S1).

**Fig. 4:**
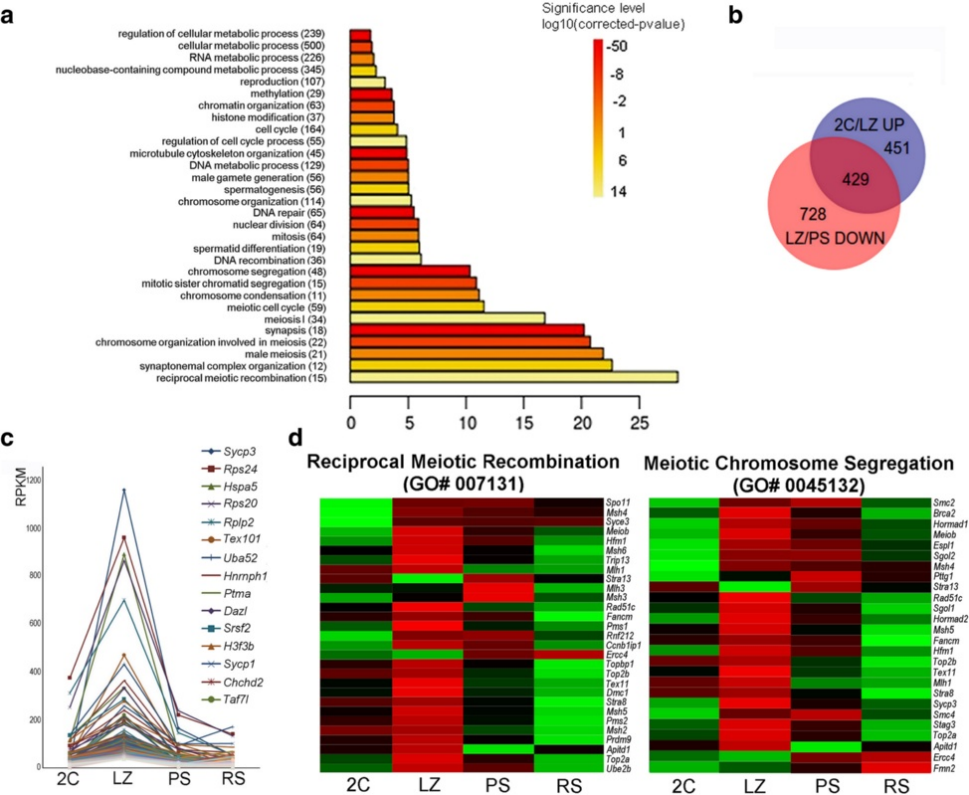
Enriched GO categories and differential expression of genes in early meiotic prophase (LZ). **a**. Enrichment analysis of biological process GO terms of up-regulated genes in 2C/LZ comparison. The fold enrichment shows the ratio of observed *vs* expected genes for each category, with an adjusted *p*-value <0.01. **b**. Venn diagram showing separate and overlapping expression between comparisons of the lists “2C/LZ up” (*i.e*. genes that are up-regulated in LZ compared to 2C) and “LZ/PS down” (*i.e*. genes that are down-regulated in PS compared to LZ). The intersection contains a subset of DEG whose expression peaks in early meiotic prophase. **c**. Graphical representation of the expression levels (RPKM) of the genes within the LZ peak in the four cell populations. The fifteen top genes are listed to the right in decreasing order according to their expression levels in LZ. **d**. Heat maps showing relative expression levels of the genes contained within GO categories “reciprocal meiotic recombination” (GO# 0007131) and “meiotic chromosome segregation” (GO# 0045132). High expression levels are indicated in red and low expression levels in green

To understand the underlying biological processes in the LZ cell population, we carried out gene ontology (GO) analysis of the DEG. For up-regulated genes, terms related to cellular metabolism, RNA and DNA metabolic processes, reproduction, chromatin organization, cell cycle, spermatogenesis, microtubule cytoskeleton organization, DNA repair, mitosis, meiosis, meiotic recombination, chromosome condensation and segregation, and synaptonemal complex, were among the most significant (*p* < 0.01) (Fig. 4a). On the other hand, down-regulated genes from 2C to LZ mostly fell into organ and tissue development-related, and extracellular matrix and cell adhesion-associated categories (*p* < 10^−7^). Besides, genes with shared expression between 2C and LZ (*i.e*. those that remained fairly constant in both lists, −2 < FC < 2), were mostly related to translation, intracellular transport, mRNA processing, cellular macromolecule localization, generation of metabolites and energy, chromatin organization, cellular respiration, and other general metabolic processes (*p* < 10^−5^). Thus, meiosis-related GO terms show significant enrichment during early meiotic prophase and not before.

Nearly half of the genes that were up-regulated in LZ (429) showed an expression peak, as they decreased before P (Fig. 4b and c). This expression peak is illustrated by temporal expression profiles 2 and 7 in Fig. 3c. GO analysis showed that this subgroup of genes (unique or differential of LZ stages) basically fell within the same categories as the whole group of up-regulated genes in the 2C/LZ comparison (*p* < 0.01; Additional file 1: Figure S2). This allows to define a set of genes whose function is related to meiotic processes, among others, that down-regulate before the P stage. Interestingly, the gene list in the LZ peak included genes whose protein products are still observed or are known to be active beyond LZ stages (*e.g. Top2a* [45], *Scml2* [46], and many others) and, in some cases, even during spermiogenesis (Additional file 2: Dataset S1). Examples of the latter are the genes coding for sperm surface-heat shock protein HSPA5 [47], TEX101 [48] (for the latter two see also Fig. 4c) and its interacting proteins DPEP3 [49] and LY6K [50], to name a few. For TEX101 and LY6K, their requirement for sperm migration into the oviduct and male fertility have been shown [51, 52].

In order to exemplify the high number of genes that show an expression peak during the short LZ stages, we selected two GO categories: “reciprocal meiotic recombination” (GO# 0007131) and “meiotic chromosome segregation” (GO# 0045132), which take place during the P stage and anaphase, respectively. All the genes within these two GO categories were clustered according to their expression values. As can be seen from the heat maps in Fig. 4d, genes in both categories were largely turned on in LZ; moreover, the expression of most genes dramatically decreased at the P stage.

### The pachytene transcriptome reveals widespread early expression of genes related to postmeiotic processes

**Fig. 5:**
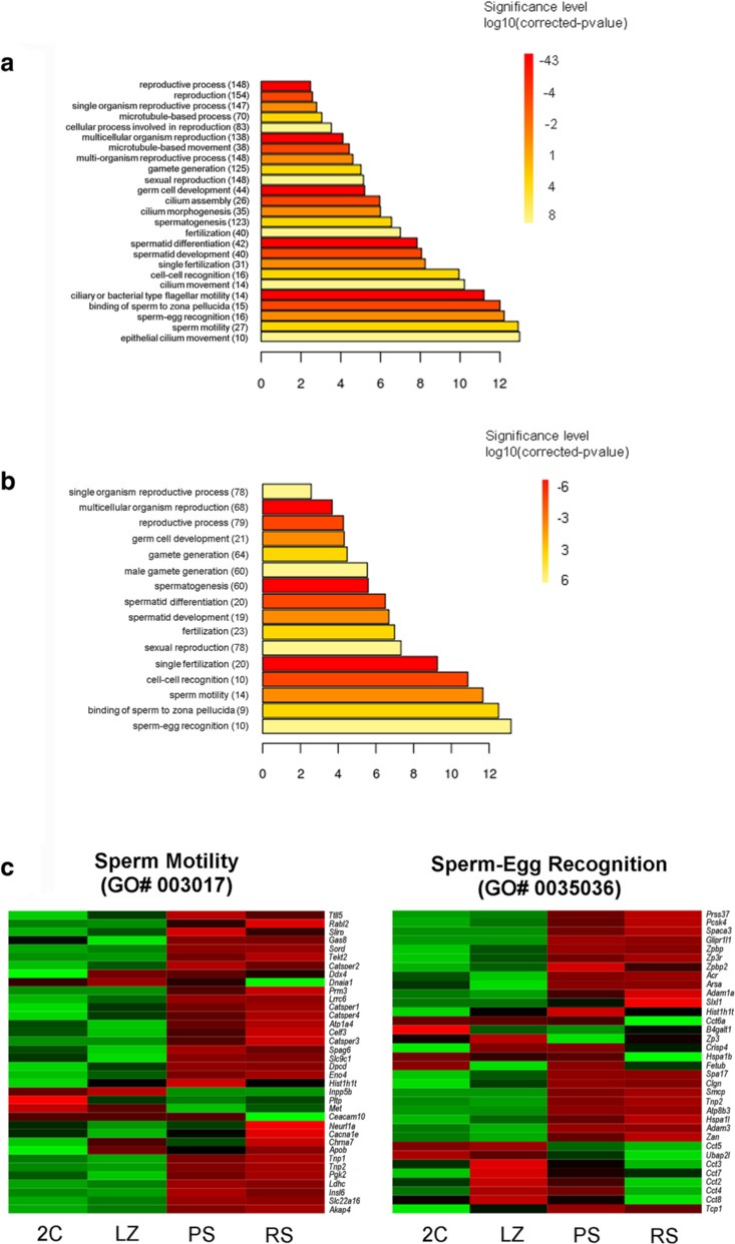
Enriched GO categories and differential expression of genes in PS and RS. **a**. Enriched biological process GO terms of up-regulated genes in the PS population compared to LZ. **b**. Enriched GO terms of up-regulated genes in RS compared to PS. **c**. Heat maps of the GO categories “sperm motility” (GO# 003017) and “sperm-egg recognition” (GO# 0035036). High expression levels are indicated in red and low expression levels in green

GO analysis of the up-regulated genes in PS compared to LZ (LZ/PS) showed enrichment in completely different biological processes. Terms related to reproduction, spermatogenesis, gamete generation, spermatid differentiation and development, fertilization, cilium and flagellum assembly and motility, sperm-egg recognition, and binding of sperm to zona pellucida, were among the most significantly represented GO categories (*p* < 0.01; Fig. 5a). Basically, the same GO terms were enriched in PS/RS (*p* < 0.01; Fig. 5b). In this regard, when pathway analysis was performed for each set of DEG, a considerable coincidence (three out of five) was found between PS and RS top canonical pathways, while no overlaps were found with LZ. Besides, four of the top five molecular and cellular functions were shared between PS and RS, while only two were in common with LZ (Additional file 3: Dataset S2).

It is worth mentioning that the number of genes within each of the above-referred GO categories was higher for LZ/PS than for PS/RS comparison (see Fig. 5a and b), which indicates that more genes related to spermiogenesis-associated processes are up-regulated in PS than in RS.

In order to exemplify the early expression of spermiogenesis-and fertilization-related genes, two processes that reflect late postmeiotic events were chosen for further analysis: sperm motility (GO# 003017) and sperm-egg recognition (GO# 0035036). As observed in Fig. 5c, the vast majority of the genes included in both categories - whose role is related to the mature spermatozoa - are activated at the P stage.

### Validation by qRT-PCR

To validate the RNAseq data, we selected 13 genes representative of the different expression profiles according to RNAseq results, and examined their expression levels *via* qRT-PCR in triplicates (three biological replicas for each cell population). In particular, we chose a gene representative of the 2C population (*Col1a1*), four genes that reflected the LZ expression peak (*Sycp3*, *Dazl*, *Tex15*, and *Top2a*), and eight genes that up-regulate in the PS cell population (*Dnahc8*, *Tcte3*, *Atp8b3*, *Clgn*, *Spa17*, *Ldhc*, *Tnp1*, and* Prm1*). With regard to the PS cell population, we deliberately selected genes that appeared as up-regulated in spermatocytes in our RNAseq study but whose protein products are known to be related to late post-meiotic functions: CLGN (calmegin) is a chaperone required for sperm-egg interaction [53]; LDHC (lactate dehydrogenase c) and DNAHC8 (dynein, axonemal, heavy chain 8) are related to sperm motility [54, 55]; ATP8B3 (ATPase, aminophospholipid transporter, class I, type 8B, Member 3) is involved in acrosome development and/or fertilization [56]; SPA17 (sperm autoantigenic protein 17) plays a role in sperm maturation, capacitation, acrosomal reaction, and interactions with the oocyte zona pellucida [57]; and TNP1 (transition protein 1) and PRM1 (protamine 1) are constituents of the chromatin of elongating spermatids and sperm, respectively [58, 59]. *Ppp1cc* and *Tax1bp1* were selected as control genes for normalization [60, 61] (see Additional file 2: Dataset S1).

**Fig. 6:**
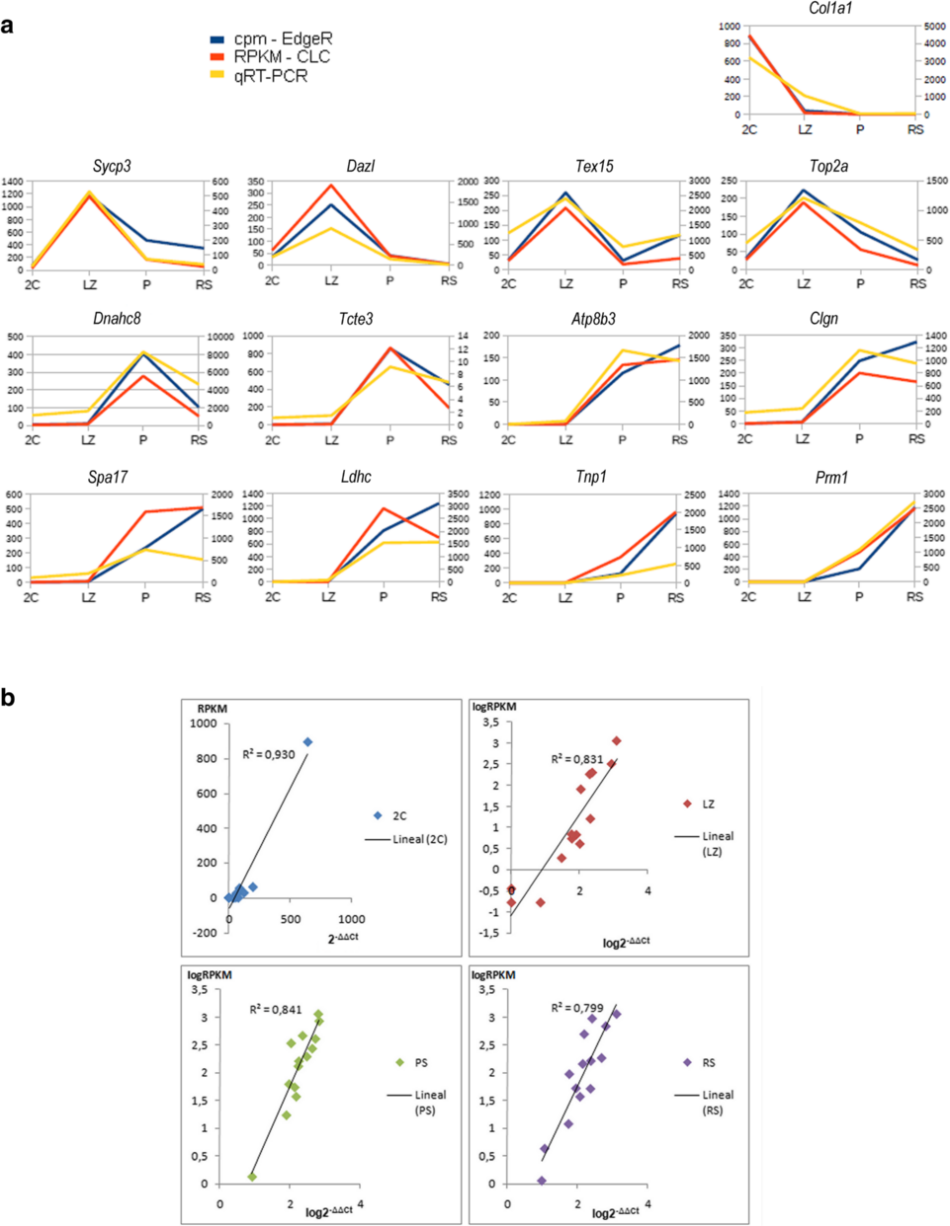
Dynamic expression patterns of 13 selected genes representative of the different expression profiles. The analyses were carried out by RNAseq (CLC bio and edgeR) and qPCR. **a**. Expression profiles obtained by RNAseq analysis (CLC bio: orange; edgeR: blue) and qPCR analysis (yellow). *Col1a1*: *collagen*, *type I*, *alpha 1*; *Sycp3*: *synaptonemal complex protein 3*; *Dazl*: *deleted in azoospermia*-*like*; *Tex15*: *testis expressed 15*; *Top2a*: *DNA topoisomerase II*, *alpha isozyme*; *Dnahc8*: *dynein*, *axonemal*, *heavy chain 8*; *Tcte3*: *T*-*complex*-*associated*-*testis*-*expressed 3*; *Atp8b3*: *ATPase*, *aminophospholipid transporter*, *class I*, *type 8B*, *member 3*; *Clgn*; *calmegin*; *Spa17*: *sperm autoantigenic protein 17*; *Ldhc*: *lactate dehydrogenase c*; *Tnp1*: *transition protein 1*; *Prm1*: *protamine 1*. **b**. Correlation between the expression levels in RNAseq analysis (RPKM values of CLC bio analysis) and those obtained by qPCR analysis for the 13 selected genes in the four testicular cell populations

The dynamic expression patterns of all genes in the four cell populations were consistent with both RNAseq analyses (CLC bio and edgeR) (Fig. 6a). Additionally, there was a high correlation between RNAseq and qRT-PCR data (Pearson r^2^ between 0.81-0.93 for CLC bio -Fig. 6b, and 0.73-0.93 for edgeR-derived results), thus supporting the reliability of our RNAseq data.

Besides, previous studies on the expression patterns for some of the selected genes were useful to further validate our results [56, 62, 63].

### X chromosome gene expression signatures during meiosis

Some peculiar aspects concerning the X chromosome in males have been reported. On one hand, a set of reports have indicated that in mouse the X chromosome is significantly enriched in spermatogonia-specific genes [64, 65] and impoverished in genes expressed during late spermatogenic stages [65]. In addition, it is acknowledged that in mammals the X chromosome is transcriptionally silenced during meiotic prophase I. This process, known as meiotic sex chromosome inactivation (MSCI), has been proposed to avoid recombination between non-homologous regions of the sex chromosome pair [66]. As the availability of highly purified spermatogenic cell populations enabled to follow these processes in detail, we used the above mentioned features to cross-check our data with previously published results.

Concerning gene distribution, we observed a significant enrichment on the X chromosome for protein-coding genes that are differentially expressed previous to MSCI (*i.e*. 2C and LZ peak; *p* < 5 × 10^−5^). A moderate enrichment (*p* < 0.05) for RS-specific genes was evidenced as well (in agreement with [38]), while an important underrepresentation on the X chromosome was found for the whole set of genes that are up-regulated from P onwards (*i.e*. PS and RS together; *p* < 5 × 10^−4^). This is in accordance with a previous work that identified an abundancy of early spermatogenic genes and a scarcity of late genes on the X chromosome of *Spo11*^−/−^ mice, in which meiosis is blocked before the P stage [65].

**Fig. 7:**
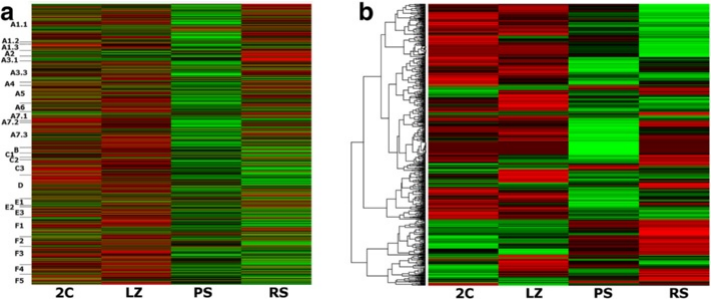
Heat maps showing relative expression levels of X-linked protein-coding genes in the four cell populations. **a**. The genes were ordered according to their position on the chromosome from *p* to *q*. Chromosome bands are indicated to the left of the figure. **b**. Hierarchical clustering. High expression levels are indicated in red and low expression levels in green for both heat maps

Regarding MSCI, we analyzed the expression of all X chromosome-linked protein coding genes present in our RNAseq data (812 genes). We generated two heat maps representing the expression levels in the four populations: a first one ordered according to the position of the genes on the chromosome from *p* to *q* (Fig. 7a), and a second one showing their hierarchical clustering (Fig. 7b). This allowed to observe the inactivation of the X chromosome, which further validates our data. As previously reported in a microarray study [35], we detected a massive switch off of X-linked genes between LZ and PS cell populations (see Fig. 7a). Interestingly, we identified a cluster of ~70 genes that showed an opposite behavior to that of most X-linked genes, as they escaped MSCI being up-regulated in PS (see Fig. 7b). Nearly half of these genes showed a 5-to 1000-fold increase from LZ to PS, and were clearly identified as differential of PS in our comparative analysis (Additional file 4: Dataset S3). Among those genes with a known function, many code for sperm-related proteins such as AKAP4 that is involved in sperm motility [67], CYPT1 that is a sperm-specific component of the post-acrosomal perinuclear theca [68], CYLC1, which forms part of the sperm head cytoskeleton, and SPACA5, a sperm acrosome-associated protein, among others. Besides, five predicted proteins contain a H2A conserved domain, and are putative histone cluster 2 family members.

The heat map displayed according to gene position on the chromosome also evidences the partial transcriptional reactivation of X-linked genes in RS. Surprisingly, reactivated genes were not scattered along the X chromosome but mostly concentrated on the centromere-nearest chromosome half (see Fig. 7a).

## Discussion

### Advantages of the experimental approach

Few studies on differential gene expression along mouse spermatogenesis by means of NGS have been published up to now [11, 38–40]. All of them employed either whole testis or stage-specific enriched cell populations where the purity of the fractions was not optimal for a detailed analysis of gene expression patterns. Besides, most previous reports using enriched cell populations (including NGS [38, 41] and most microarray studies) have viewed PS as the representative meiotic prophase stage, and therefore it has not been possible to discriminate between transcripts synthesized for the first time in PS and those originated during earlier meiotic prophase stages.

Dealing with the technical limitations described above, we have incorporated some key methodological improvements thus significantly contributing to the reliability of the results: i) the fast method for the preparation of the testicular cell suspensions [29, 30], and the use of VDG [27] that is a vital dye excitable at 488 nm, both help preserve the integrity of the RNAs; ii) the flow sorting protocol used here enabled to obtain all the testicular cell populations at very high purity rates, allowing gene expression studies with negligible cross contamination. As a consequence, we can assert that the results from the RNAseq studies reflect differential expression in each of the cell types to an unprecedented accuracy level (see also below); iii) the availability of a highly purified LZ fraction (identified as a discrete cell population by FCM) for RNAseq analysis allowed to assess the temporal expression profile along key meiotic prophase I stages, in contrast to the use of whole testes from animals of increasing ages that permits to ascertain when genes are switched on, but not their switch off time point. Here, the gene expression pattern of LZ spermatocytes was compared with that of PS, thus enabling to follow the up-and down-regulation of the different genes.

All the above mentioned improvements allowed us - to our knowledge for the first time - to reliably relate stage-specific gene expression signatures (GES) with the processes that take place at the different spermatogenic stages.

### Main contributions to the knowledge of gene expression patterns along spermatogenesis

An important conclusion from the analysis of GES in this work is that a good deal of genes involved in early as well as late meiotic processes are already on at early meiotic prophase (*i.e*. LZ). As the GO enrichment analysis of the transcripts whose expression levels remain fairly constant between 2C and LZ cell populations mostly rendered terms associated with general processes and very few meiosis-related ones, we can conclude that most meiosis-related genes that are expressed in LZ, are up-regulated at the beginning of meiosis. Remarkably, a high number of these genes are only on for the short time lapse of LZ (2–3 days), being down-regulated before P. Therefore, the temporal expression pattern of many meiotically-expressed genes is evidenced as a sharp LZ peak. More intriguingly, a small group of genes whose products are known to be present during late spermiogenic stages were also contained within the LZ peak set.

**Fig. 8:**
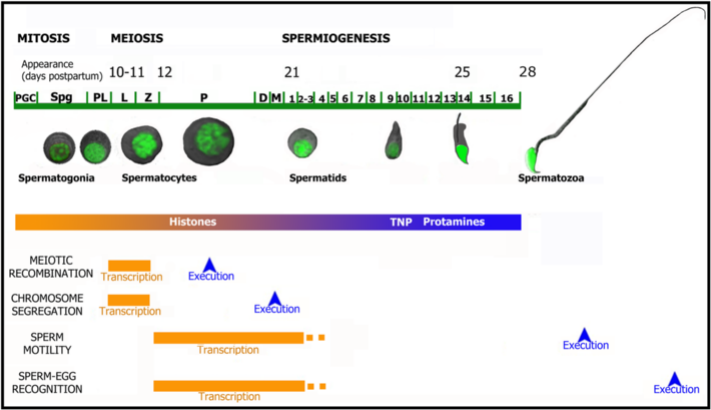
Representation of the transcription and execution times of four selected GO categories. The diagram represents the time when the biological processes shown in the heat maps in Figs. 4c and 5c are transcriptionally activated, and when these processes are executed along the first spermatogenic wave in mouse. The onset (in dpp) for the different stages along the first spermatogenic wave is denoted on top. The time of histone substitution - first by TNP and then by PRM - is also represented. PGC: primordial germ cells; Spg: spermatogonia; PL: preleptotene; L: leptotene; Z: zygotene; P: pachytene; D: diplotene; M: meiotic divisions

The two GO categories we chose for exemplifying the early transcription of meiotic genes, which represent meiotic prophase (“reciprocal meiotic recombination”) and late meiotic events (“meiotic chromosome segregation”) respectively, are illustrated in Fig. 8. Noteworthy, there is a gap between the time when these programs are transcriptionally activated, and the time when they are executed. Moreover, as stated above, many of the implicated genes are down-regulated before P. A logical conclusion of the aforementioned is that these transcripts must be mostly translated in LZ (*i.e*. before their degradation) and, for those proteins whose product remains present/active beyond LZ, the protein should be kept for longer. In this regard, a recent proteomic study described different regulation mechanisms for the dynamic gene expression changes that take place during spermatogenesis [69]. Interestingly, one of those mechanisms, which the authors termed “transcript degradation”, refers to genes whose mRNA levels drop from spermatogonia to PS while their protein levels remain relatively constant until marked for degradation at a later stage. Despite the facts that only a limited number of proteins were identified in that study, and that it did not include the LZ stages, “transcript degradation” would probably describe the regulatory mechanism for many genes within the LZ peak.

Another striking result is the massive change in GES at the P stage, which we named as “the pachytene switch”. The general profile of up-regulated and down-regulated genes was more similar between 2C and LZ cell populations on one side, and amid PS and RS on the other. These findings are in agreement with a couple of microarray studies reporting a global expression switch in the testicular transcriptome during the progression from Z to P [10, 35]. However, we have demonstrated here for the first time that the genes which turn on during the P switch are mostly related to postmeiotic processes (see below) and not to meiosis, as previously thought (*e.g*. [10]).

Surprisingly, the number of DEG in RS was lower than that of PS, thus contradicting some previous microarray studies [7, 34]. Moreover, almost half of the up-regulated genes in RS (FC ≥ 2) were previously identified as up-regulated in LZ/PS. This supports the idea that the biggest change in gene expression patterns takes place at the P stage (*i.e*. during meiotic prophase I) and not at the beginning of spermiogenesis as previously stated. Besides the fact that microarray analyses of spermatogenic transcriptome were partial, the different results could be partly due to the low sensitivity of the microarray technique in comparison to RNAseq [70].

We found that the P switch is accompanied by an overall change in the involved biological processes, indicating that - in terms of differential gene expression - LZ and PS are committed to the transcription of different spermatogenic programs, despite both being primary spermatocytes. Interestingly, while LZ spermatocytes differentially express a high number of meiotic genes, PS mostly turn on spermiogenesis-related genes, thus revealing an apparent inconsistency between the meiotic role of the PS - which reflects its protein content, largely related to meiotic recombination - and its differential mRNA profile. In accordance with this, when we selected two GO categories indicative of processes that take place in mature sperm (“sperm motility” and “sperm-egg recognition”) for further analysis, the gap between the transcriptional activation time of the genes within both categories (mostly the P stage) and the execution time of these processes was evident, as illustrated in Fig. 8. Moreover, qRT-PCR results allowed to confirm the early turn on during the P stage of some selected genes whose products are known to be required for sperm functionality.

The existence of translational delays in spermatogenesis is well known [58, 71], and has been associated to the design of a strategy to regulate protein synthesis in cells that do not transcribe, as transcription in elongating and elongated spermatids is silenced after sequential histone replacement first by transition proteins (TNP) and then by protamines (PRM) [58, 59]. Translational repression has been adopted as a strategy to regulate the time of synthesis for proteins whose production in earlier spermatogenic stages (*e.g*. RS) would be detrimental (*e.g*. [58, 72]). Besides, post-transcriptional regulation mechanisms are acutely sensitive and can respond fast to environmental changes, as required in reproductive systems [73]. The mechanisms employed to achieve these unusually high levels of post-transcriptional regulation may involve mRNA sequestration as free ribonucleoprotein particles (RNPs) [8], binding of repressor proteins to UTRs of testis-specific transcripts (*e.g*. [74]), regulation of poly (A) tails length [71], use of upstream repressor open reading frames (uORFs) [75], participation of microRNAs, piRNAs [76], and antisense long non-coding RNAs (lncRNAs) [69, 77], and retention of RNAs in the chromatoid body [78], among others.

Although all the above-mentioned regulatory mechanisms have been extensively studied in spermatogenic cells, it is generally accepted that most transcripts whose protein products are required in elongating/elongated spermatids are transcribed by RS [8, 34]. In spite of that, there are some reports of genes whose transcripts start to appear in spermatocytes and are translationally inactive until their protein is first detected in spermatids, such as *Pgk2* [79, 80] and others. Our findings show that the transcription of genes for spermatid- and sperm-specific proteins in PS is a much more widespread phenomenon than previously thought.

These results would be in agreement with the proteomic study by Gan et al. [69], who compared their data with publicly available transcriptomic information from microarray studies. They reported that of 253 proteins with high expression in RS and elongated spermatids, 54 % were translationally repressed in PS, 60 % of which were derepressed later on in RS. Although important spermiogenesis-related proteins are missing in Gan’s list (*e.g*. PRM, TNP), we contrasted our transcriptomic results with the expression data of their spermatid-specific cluster. Interestingly, according to our RNAseq data, 67 % of the proteins from that cluster appeared as translationally repressed in PS, 70 % of which were derepressed in RS (Additional file 5: Dataset S4). LDHC [54], AKAP4 [67], and CLGN [53], are examples of proteins within this category. In view of these results, it is tempting to speculate that the great length of the P stage in all the studied metazoan species could be related, at least in part, to the partial turning off of the meiotic gene expression program, and the turning on of the spermiogenic one.

A particularly surprising outcome concerned the PRM and TNP. It has been long stated that their mRNAs in mouse, rat and human start to be transcribed in RS [81–85] and remain repressed until the stages of elongating spermatids, when they are translated [86, 87]. This means that in mouse, for example, this repression mechanism would be operative for up to a week. The mechanisms for post-transcriptional regulation of these mRNAs have been extensively studied [88], and it is known that abnormal *Prm* and *Tnp* expression including premature translation causes spermiogenesis arrest and infertility (*e.g*. [72, 89, 90]). Interestingly, in this work we found that although RS showed the highest *Prm* and *Tnp* mRNA levels among the four analyzed cell populations, *Prm* and *Tnp* genes were turned on and exhibited significantly high mRNA levels as early as during the P stage, especially *Prm1*, *Tnp1* and *Tnp2* (see Additional file 2: Dataset S1). This was confirmed by qRT-PCR for *Prm1* and *Tnp1*. Moreover, *Prm1*, *Tnp1* and *Tnp2* were among the 20 top highly expressed transcripts in PS. Thus, our findings place the switching on of *Prm* and *Tnp* in mouse between one and two weeks earlier than previously thought, and indicate that the repression mechanisms that regulate their translation time must be active for much longer than anticipated. Remarkably, an early work by Iatrou et al. [91] had already reported the presence of *Prm* mRNA in primary spermatocytes in the rainbow trout by quantifying *Prm* cDNA-RNA hybrid formation.

Another interesting result was obtained in relation to *Dazl* (*deleted in azoospermia*-*like*), which encodes a germ cell-specific RNA-binding protein that is required for the differentiation of germ cells in vertebrates [92]. Controversies exist concerning the expression pattern of *Dazl*. While it has been reported that in adult mice *Dazl* expression is restricted to premeiotic stages [93], other studies have suggested its expression all through germ cell development, with high levels in PS [94]. Our NGS results, confirmed by qRT-PCR, show that *Dazl* exhibits a sharp LZ transcription peak. This fact remained unnoticed until now, probably due to the lack of expression studies using isolated LZ cell populations. This result is consistent with the finding that DAZL binds *Sycp3* and *Mvh* (*mouse vasa homolog*, also known as *Ddx4*) mRNAs *in vivo* [95, 96], both of which are up-regulated in LZ as well (see Additional file 2: Dataset S1).

Finally, we decided to use our RNAseq data in order to analyze some particular spermatogenesis-related aspects on the X chromosome and compare our results with previously reported ones. In this regard, we have been able to follow MSCI on heat maps. A massive inactivation was observed between LZ and PS in agreement with previous results [35]. Moreover, we detected an important enrichment on the X chromosome for genes expressed during early spermatogenic stages, which is also coincidental with published reports (*e.g*. [64, 65]). Interestingly, our results also disclose some novel insights regarding X chromosome behavior during spermatogenesis. First, the availability of highly pure LZ cell populations for direct comparison with PS allowed us to identify a cluster of genes that - contrary to the general trend - are up-regulated in PS. In this connection, although protein-coding genes that escape MSCI have been found in the domestic dog and some evidence suggests that MSCI might not be totally complete in humans as well [97], so far no X-linked mRNA genes had been reported to escape MSCI in mouse [98]. Many of the murine X-linked genes that appear as up-regulated in PS in our list, code for proteins that are functional in elongated spermatids and sperm. This would be in agreement with the massive switch on of spermiogenesis-related genes in PS reported above, and suggests that the expression of these genes must be subject to a specific epigenetic regulation that allows avoiding MSCI due to their importance for the development of healthy sperm. Besides, a number of X-linked genes coding for predicted histone H2A-family members also appeared as up-regulated in PS. Several testis-specific H2A variants are known to be involved in chromatin dynamics in spermatids by assisting in the displacement of histones by TNP [99, 100]. Moreover, some of them remain stably associated with the genome in mature sperm [101]. We speculate that these X-linked H2A genes that are activated in PS, escape MSCI because they would be somehow required for chromatin reorganization in spermatogenic cells.

Another surprising finding was the differential activation of gene expression along the X chromosome in RS, with a notably higher number of up-regulated genes on the centromere-nearest chromosome half. This suggests undisclosed mechanisms of epigenetic regulation for transcriptional reactivation in RS. Interestingly, a similar reactivation pattern has been observed for the dog [97].

## Conclusions

This work represents an important advance regarding testicular transcriptome analyses in mouse. First, RNAseq technology was combined for the first time with a powerful flow cytometry-based protocol to sort highly pure spermatogenic cell populations, which allowed to obtain information about stage-specific gene expression with unprecedented reliability. Second, transcriptome profiling by RNAseq for early prophase meiocytes is reported here for the first time. Using the above-mentioned improvements we disclosed here the following novel results: a) an important proportion of the meiotic program is already on at early meiotic prophase; b) a considerable number of genes have a marked expression peak at LZ stages, their transcripts being present for a very short time lapse; c) there is a massive change in GES at the P stage; d) the largest number of genes related to spermiogenesis and sperm functionality are turned on in PS, thus revealing a higher incidence of post-transcriptional regulation in spermatogenesis than previously stated; e) an important part of differential gene expression in RS corresponds to the up-regulation of genes which turn on earlier, during the P stage; f) a cluster of X-linked genes in mouse are up-regulated in PS, thus escaping MSCI.

Taken together, our data provide a highly reliable information set about gene expression in purified spermatogenic stage-specific cell populations including early meiotic prophase, for further studies on the molecular bases of male reproduction in mammals.

## Methods

### Animals

Male CD-1 Swiss mice (*Mus musculus*) at different ages (10–11 and 24–25 dpp) were obtained from the animal facility at Instituto de Investigaciones Biológicas Clemente Estable (IIBCE, Montevideo, Uruguay). Flow sorting was performed on testicular cell suspensions of pooled material from a dozen pups aging 10–11 dpp and three to four mice aging 24–25 dpp per assay. Animals were euthanized by cervical dislocation (see Ethics Approval below).

### Preparation of cellular suspensions

Testicular cell suspensions were prepared by a procedure previously described in our laboratory [27, 29]. Reagents and plasticware were handled in RNAse-free conditions. Briefly, testes were dissected into 35 mm glass Petri dishes containing ice-cold separation medium (10 % v/v fetal calf serum in Dulbecco’s Modified Eagle’s medium [DMEM], with high glucose and L-glutamine), and cut into 8–10 mm^3^ pieces after removal of the tunica albuginea. Between three and four of these pieces per round were immediately placed into a sterile disposable disaggregator unit (Medicon; Beckton-Dickinson [BD], CA) plus 1 mL of ice-cold separation medium, and processed for 30 sec in the Medimachine System (BD), an automated electro-mechanical solid-tissue disaggregator. The resulting cell suspension was recovered from the Medicon unit with a 5 mL disposable syringe, sequentially passed through two 50 μm Filcon filter units (BD), and placed on ice. Cells were counted by means of a Neubauer chamber and resuspended at a concentration of up to 3–5 × 10^6^ cells/mL in separation medium. NDA (2-naphthol-6,8-disulfonic acid, dipotassium salt; Chemos GmbH, Regenstauf, Germany) was added to the suspension to a final concentration of 0.2 % (w/v) in order to prevent cell clumping.

### Cell analysis and sorting by flow cytometry

Mouse testicular cell suspensions were analyzed and sorted following the method earlier described by Rodríguez-Casuriaga et al. [27]. In brief, cell suspensions were stained for 1 h at 37° with VDG (Invitrogen-Life Technologies, Carlsbad, CA) at a final concentration of 10 μM. Samples were analyzed and sorted with a FACSVantage flow cytometer (BD) furnished with an argon ion laser (Coherent, Innova 304) tuned at 488 nm of excitation wavelength (100 mW). A 70 μm nozzle was selected to perform FCM analysis and cell sorting. Fluorescence emitted from VDG was collected in the FL1 channel using a 530/30 band pass filter. To optimize fluorescence detection, instrument linearity, and doublet discrimination performance, DNAQC particles (BD) were used. CELLQuest software (BD) was applied to analyze the following parameters: forward scatter (FSC-H), side scatter (SSC-H), pulse-area or total emitted fluorescence (FL1-A; VDG fluorescence intensity), and pulse-width or duration of fluorescence emission (FL1-W). Doublets were excluded using dot plots of FL1-A *vs* FL1-W. Sorting regions for 2C, LZ, PS and RS were determined on VDG fluorescence intensity *vs* FSC-H dot plots.

The 2C and LZ cell populations were classified from testicular cell suspensions of individuals aging 10–11 dpp, while the PS and RS cells were obtained from 24–25 dpp ones (different animal pools were used to obtain each cell population in order to avoid paired samples for NGS analysis). Cells were sorted at a rate of 500–1,000/sec. Sorting mode was set in Normal-C, and 3 sorted drops as envelope were used. During the sorting procedure, sample and collecting tubes were maintained at 4° with a dedicated refrigeration unit (Lauda, Brinkmann, Delran, NJ) connected to the flow cytometer. Cells were collected into 12 × 75 mm sterile polystyrene tubes containing 0.5 mL PBS treated with 0.1 % DMPC (PBS-DMPC) and subsequently spun down (500 g, 10 min, 4°), washed with PBS-DMPC, deep frozen in liquid nitrogen, and stored at − 80°.

### Microscopical observations and immunocytochemistry

In order to determine the optimal age for LZ enrichment in the absence of PS contamination, whole testes of pups ranging from 9 to 14 dpp (sampled at one-day age differences) were fixed in 0.1 M phosphate buffer (pH 6.8) containing 2.5 % glutaraldehyde, post-fixed in 1 % osmium tetroxide, and embedded in Epon (Durcupan ACM Fluka, Sigma-Aldrich, St. Louis, MO) as previously reported [19]. 1 μm sections were cut with a Power Tome XL Ultra-microtome (Boeckeler Instruments, Tucson, AZ), stained with toluidine blue, and examined by light microscopy with an Olympus FV300 confocal microscope. Photographs were taken with an Olympus DP70 digital camera using the DPController v. 1.1.1.65 software.

To confirm the purity of each meiotic prophase sorted fraction, an aliquot of each classified fraction of 4C cells (LZ and PS) was immunolabeled with a rabbit antibody raised against the C-terminal region of mouse SYCP3 (Acris Antibodies GmbH, Herford, Germany; RA25051, 1:100) and Texas Red-tagged goat anti-rabbit secondary antibody (Abcam, Cambridge, MA; ab6719, 1:500) as described earlier [19]. Fluorescent images were acquired with the Fluoview v.4.3 software.

### RNA extraction and amplification

Total RNA of each sorted fraction (coming from pooled material from various specimens, as stated above) was extracted with the PureLink RNA Mini Kit (Ambion-Life Technologies, Carlsbad, CA), following the recommendations of the manufacturer. RNA quantitation was performed by fluorometry using Qubit 2.0 and RNA HS Assay (Life Technologies). In general, ~ 50–70 ng total RNA was obtained from ~ 3 × 10^5^ sorted cells. RNA linear amplification of high purity sorted cells was performed with the Ovation RNA-Seq System v2 (NuGEN, San Carlos, CA), an RNA-based single-primer isothermal amplification (SPIA) technology that has proven to be highly sensitive for whole-transcriptome sequencing using limited amounts of total RNA [43]. RNA amplification was performed following the manufacturer’s recommendations. Quality and quantity of the resulting double-stranded cDNA were evaluated by means of a 2100 Bioanalyzer (Agilent, Santa Clara, CA) and Nanodrop 1000 (Thermo Fisher Scientific, Wilmington, DE), respectively.

### RNAseq, data processing and analysis

Libraries were constructed and sequenced at Macrogen (Seoul, Korea), on Illumina HiSeq2000 platform. Quality, length trimming and RNAseq quantification were conducted using CLC Genomics Workbench 6.5 (CLC bio, http://www.clcbio.com). Low quality reads (Q < 33, *p* ≤ 0.01) and the first 15 bases of the reads with a distinctly non-random base composition were also removed according to [102]. CLC Genomics Workbench 6.5 was used for all data analysis downstream.

High-quality reads for each cell population (2C, LZ, PS, RS) were aligned to the *Mus musculus* genome GCRm38 assembly from C57BL/6 J strain, using the RNAseq pipeline from CLC bio. Paired-end Illumina RNAseq data were mapped with the following parameters: a) maximum number of allowed mismatches was two; b) minimum length and similarity fraction was set at 0.9; and c) minimum number of hits per read was 10. Gene expression values were reported as RPKM, as described by Mortazavi et al. [103].

In order to ensure comparability, RPKM values were normalized by quantile method implemented in CLC bio. A gene was considered as expressed if it had more than 10 aligned reads and RPKM ≥ 2. Differential gene expression between the four testicular cell populations was obtained by pairwise comparisons in chronological order of appearance along the first spermatogenic wave (2C *vs* LZ; LZ *vs* PS; PS *vs* RS). An absolute FC of 2 was used to filter the DEG. The *p* value cut-off was set at *p* ≤ 0.01 based on Kal’s Z test statistical analysis [104].

The obtained results were also analyzed using a different pipeline to verify the general output of CLC pathway. Reads that passed quality control for each cell population were aligned to the mouse genome by TopHat 2.0.4 [105]. Aligned reads were counted by HTSeq 0.6.0 [106], and differential gene expression analyses were performed with edgeR 3.2.4 [107, 108]. The biological coefficient of variation (BCV) was set manually at 0.4 as recommended in user guides for no replicates.

Based on the differential gene expression analysis among the four cell populations, we used STEM (Short Time series Expression Miner, http://www.sb.cs.cmu.edu/stem/) in order to cluster gene expression patterns in short time series. The input data file contains the time series of gene expression values (RPKM). We selected the Stem Clustering Methods based on units of change (c) defined as c = 2. Basically, if c = 2 between successive time points, a gene can go up either one or two units, stay unchanged, or go down one or two units. From these possible expression profiles, a set of candidate profiles (m) defined by the user as m = 50 were organized in such a way that the minimum distance between any two profiles was maximized. Each gene was assigned to the closest profile using a Pearson correlation based on metric distance. To determine a significance level for a given cluster, a permutation-based test was used to quantify the expected number of genes that would be assigned to each profile if the data were randomly generated. Data was considered significantly different at *p*-value = 0.05.

Webgestalt (http://bioinfo.vanderbilt.edu/webgestalt/) and David Bioinformatics Resources 6.7 (http://david.ncifcrf.gov/) were used for functional enrichment analysis of the differentially expressed filtered gene lists toward KEGG pathways and GO categories. Heat maps and barplots comparing the differential gene expression and enrichment of GO categories were produced with R bioconductor (http://cran.r-project.org/). Color gradient from red to yellow in the barplots corresponds to increasing *p*-values: red indicates low *p*-values (high enrichment), while yellow indicates high *p*-values (low enrichment). Analyses of top canonical pathways and molecular and cellular functions were performed with QIAGEN’s Ingenuity Pathway Analysis (www.qiagen.com/ingenuity).

For heat maps representing the expression levels of genes located on the X chromosome, a list of X-linked protein-coding genes was obtained from Ensembl Biomart search engine (http://www.ensembl.org/biomart/), combined with our RNAseq data for the four populations, and represented graphically through R bioconductor as mentioned above. To determine whether DEG had preferential chromosome location, hypergeometric tests were performed in R bioconductor and enrichment/depletion *p*-values were calculated.

### Confirmative qRT-PCR

For confirmative qRT-PCR assays, cells (2C, LZ, PS, and RS) were sorted from the same regions determined on VDG fluorescence intensity and FSC-H as described above. The number of collected cells was assessed by employing the CloneCyt Plus software (BD) in the Counter mode, and 3,000 cells were assayed in each reaction. Cell lysates were used for RT-PCR by means of the Power SYBR Green Cells-to-Ct Kit (Ambion-Life Technologies) essentially as instructed, using random primers. For the real-time PCR step we used 4 μL cDNA in a 25 μL reaction mix, according to the manufacturer’s instructions. All the reactions were performed in three biological replicas in a CFX96 Touch Real-Time PCR Detection System1 (BioRad, Hercules, CA). *Ppp1cc* (*protein phosphatase 1, catalytic subunit, gamma isozyme*) and *Tax1bp1* (*human T-cell leukemia virus type I-binding protein 1*) were chosen as control genes both because they were in a list of Applied Biosystem’s TaqMan endogenous controls and in a list of housekeeping genes from previous papers [60, 61]. Besides, in our RNAseq results they exhibited similar expression levels in the four assayed testicular cell populations (see Additional file 2: Dataset S1). The genes selected for confirmation by qRT-PCR are shown in Fig. 6, and all especially designed primers are listed in Additional file 1: Table S2.

Amplification efficiency was evaluated *via* standard curve analysis, and was >93 %. The average threshold cycle (Ct) was calculated for each sample using the 2^-∆∆Ct^ method normalized to *Ppp1cc*, and the results were cross-validated with *Tax1bp1*.

### Ethics approval

All animal procedures were performed following the recommendations of the Uruguayan National Commission of Animal Experimentation (CNEA, approved experimental protocol 001/02/2012). Animal housing and breeding at IIBCE was approved by CNEA (code: 008/11; http://www.cnea.org.uy/index.php/instituciones/registro/10).

### Consent for publication

Not applicable.

### Availability of data and materials

The data sets supporting the results of this article are available at the Sequence Read Archive repository, Project ID: PRJNA317251.

## Additional files

Additional file 1:
**Figure S1.** Testicular cell content evaluation in pre-pubertal mice. A. Epon-embedded cross sections of seminiferous tubules in 10–13 dpp pups, stained with toluidine blue. Bar: 50 μm. B. Analysis of meiotic prophase stages by confocal immunocytochemistry with anti-SYCP3 antibody (green) in whole testicular cell suspensions of 10–12 dpp pups. L, Z and P spermatocyte nuclei are indicated. ltZ: late zygotene. Bar: 10 μm. **Figure S2.** Enrichment analysis of biological process GO terms of genes showing an expression peak in LZ. **Table S1.** Sequencing yield of individual samples. **Table S2.** List of primers used for qRT-PCR in this study. (PDF 238 kb) Additional file 2: Dataset S1. Lists of genes and their normalized expression values in mouse 2C, LZ, PS, and RS testicular cell populations. (XLSX 570 kb) Additional file 3: Dataset S2. Top five canonical pathways and molecular and cellular functions from pairwise comparisons of the four cell populations in chronological order (LZ relative to 2C; PS relative to LZ; RS relative to PS). (XLSX 10 kb) Additional file 4: Dataset S3. List of X-linked genes showing a 5- to 1000-fold increase from LZ to PS. (XLSX 10 kb) Additional file 5: Dataset S4. Comparison of the RNAseq data generated in this work with the protein data reported by Gan et al. (2013) [69] for their cluster 4, which comprises proteins more abundant during spermiogenesis (RS and elongating spermatids) than in earlier stages. For both data sets 2C population was taken as a reference, and RNA as well as protein ratios (RATIO_m_ and RATIO_p_, respectively) between the analyzed cell populations were calculated accordingly. The quantitative relationship between RNA and protein levels was determined at the transitions 2C-PS and PS-RS (RATIO_mp_ = RATIO_m_/RATIO_p_). As in Gan’s report, RATIO_mp_ ≥ 2 at the 2C/PS transition was interpreted as translational repression, while RATIO_mp_ ≤ 0.5 at the PS/RS transition was assigned to translational de-repression. Red crosses (right column) indicate the transcripts within this group that would be translationally repressed during PS and de-repressed later on at RS. (XLSX 65 kb)

## Abbreviations

DEG: differentially expressed genes
dpp: days postpartum
FACS: fluorescence-activated cell sorting
FC: fold change
FCM: flow cytometry
FL1-A: pulse-area or total emitted fluorescence
FL1-W: pulse-width or duration of fluorescence emission
FSC-H: forward scatter
GES: gene expression signatures
GO: gene ontology
L: leptotene
LZ: lepto/zygotene
MSCI: meiotic sex chromosome inactivation
NGS: next generation sequencing
P: pachytene
PRM: protamine
PS: pachytene spermatocytes
RPKM: reads per kilobase per million mapped reads
RS: round spermatids
SSC-H: side scatter
TNP: transition protein
VDG: Vybrant DyeCycle Green
Z: zygotene
