# Supplementary material for: Transcriptome analysis of highly purified mouse spermatogenic cell populations: gene expression signatures switch from meiotic-to postmeiotic-related processes at pachytene stage
Source: BMC Genomics. 2016 Apr 19;17:294. doi: 10.1186/s12864-016-2618-1 (PMC4837615; doi:10.1186/s12864-016-2618-1)
Supplement: Additional file 1: — Figure S1. Testicular cell content evaluation in pre-pubertal mice. A. Epon-embedded cross sections of seminiferous tubules in 10–13 dpp pups, stained with toluidine blue. Bar: 50 μm. B. Analysis of meiotic prophase stages by confocal immunocytochemistry with anti-SYCP3 antibody (green) in whole testicular cell suspensions of 10–12 dpp pups. L, Z and P spermatocyte nuclei are indicated. ltZ: late zygotene. Bar: 10 μm. Figure S2. Enrichment analysis of biological process GO terms of genes showing an expression peak in LZ. Table S1. Sequencing yield of individual samples. Table S2. List of primers used for qRT-PCR in this study. (PDF 238 kb) [file 12864_2016_2618_MOESM1_ESM.pdf]

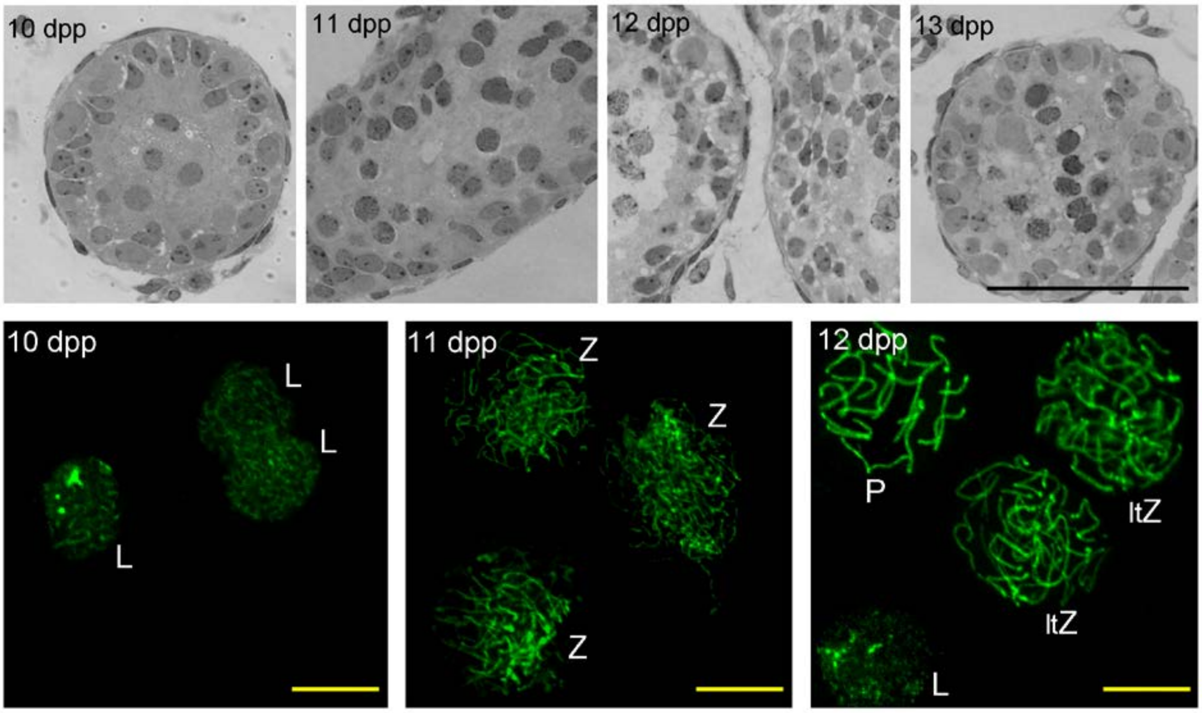

Figure S1. Testicular cell content evaluation in pre-pubertal mice. **A.** Epon-embedded cross sections of seminiferous tubules in 10-13 dpp pups, stained with toluidine blue. Bar: 50 μm. **B.** Analysis of meiotic prophase stages by confocal immunocytochemistry with anti-SYCP3 antibody (green) in whole testicular cell suspensions of 10-12 dpp pups. L, Z and P spermatocyte nuclei are indicated. ltZ: late zygotene. Bar: 10 μm.

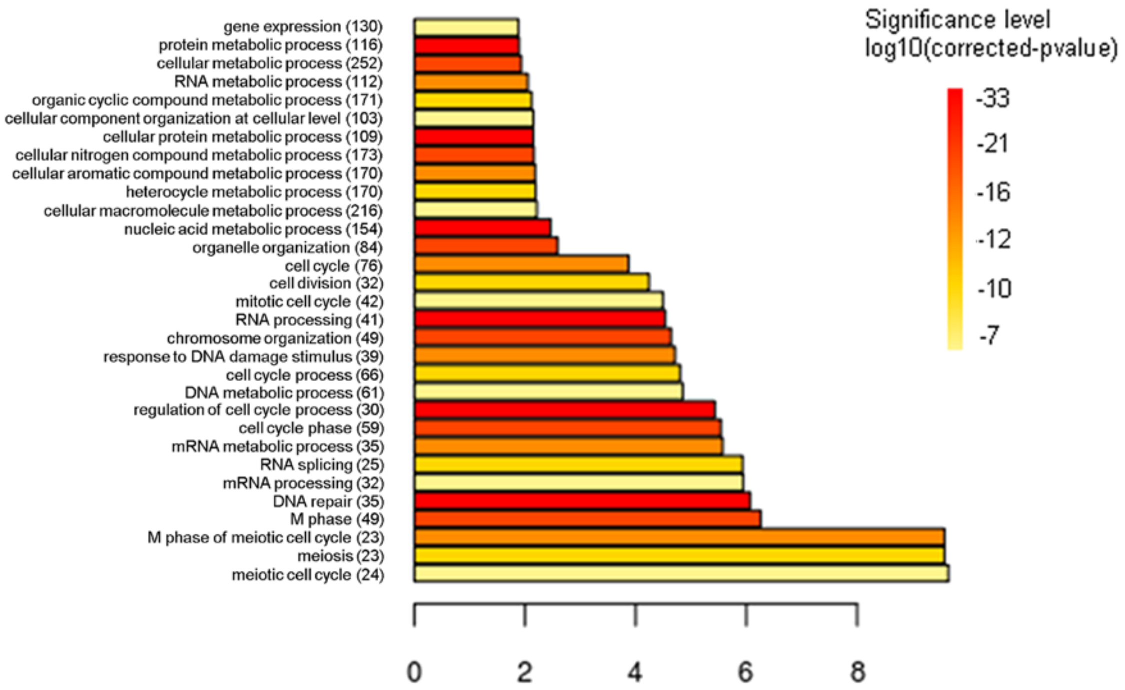

Figure S2. Enrichment analysis of biological process GO terms of genes showing an expression peak in LZ.

Table S1. Sequencing yield of individual samples.

Sample	# raw data	# reads after QC	% of aligned reads
**2C**	48,538,544	48,397,424	71.3
**LZ**	65,340,234	62,740,060	80.3
**PS**	61,988,530	59,359,696	80.2
**RS**	47,409,618	45,022,546	56.2

Table S2. List of primers used for qRT-PCR in this study.

Gene	Forward primer	Reverse primer	Product size
Atp8b3	CTCAAGGTTCTCCCCGAGAT	ACCAGGCTAACAGCTCCACA	135
Clgn	CATCAAGCCAGCCATCAGGT	GATGACAGAGCCAAGATCCC	115
Col1a1	GGTTTCCACGTCTCACCATT	CGGCTCCTGCTCCTCTTAG	116
Dazl	CTAGGCAGCCACCTCACG	TCCATCCTAACATCAATTCCTCC	240
Dnahc8	AGCTGAGGTATACGAGCATC	CAGCAGCCTGAGTGACCTAA	105
Ldhc	TCGTATCAGCGTCAACAAGG	GTGGTCGGAGTTGGAAATGT	94
Ppp1cc	CATATCTTGAGTGGTGCTTCA	GACAGCATCATCCAACGGCT	158
Prm1	GCTCACAGGTTGGCTGGCTC	TGATGGACTTGCTATTCTGTGC	251
Spa17	CTCAGAATCTCCCGTGTCAGCC	ACACTGGAACCATCCGCACC	155
Sycp3	TGTGGGGACAGCGACAGC	TACTTCACCTCCAACATCTTCA	253
Tax1bp1	AGTGTGCATTAGGAAGGTAACT	CTACGCTGAGAGGCAGTGG	176
Tcte3	TGAGTCATCAAACGGAGGTTC	AGGAAGCCTAGCATGTTCG	108
Tex15	GAAACGGATTCTGTTGCCATG	GCTTCTCATCAACCAGTCCCA	106
Tnp1	CATCACAAGTGGGATCGGTA	TCAAGAGAGGTGGAAGCAAGA	100
Top2A	TGTGTTCAACAACAGGGATTC	GAGATTCTAGTTAATGCTGCTGATA	133
